# Atypical Multifocal Motor Neuropathy Presenting With Isolated Spinal Accessory and Pectoral Nerve Involvement

**DOI:** 10.7759/cureus.111850

**Published:** 2026-06-30

**Authors:** Amna Tariq, Syed Ali Raza, Muhammad Sohaib Siddique

**Affiliations:** 1 Neurology, Hull University Teaching Hospitals NHS Trust, Hull, GBR; 2 Medical Oncology, Hull University Teaching Hospitals NHS Trust, Hull, GBR; 3 Accident and Emergency, Din Medical Complex, Burewala, PAK

**Keywords:** distal limb weakness, intravenous immunoglobulin (ivig), multifocal motor neuropathy, neurology, pectoral nerve, spinal accessory nerve

## Abstract

Multifocal motor neuropathy (MMN) is a rare, immune-mediated disorder typically characterized by slowly progressive, asymmetric distal limb weakness. Cranial nerve involvement is distinctly uncommon, and isolated involvement of the spinal accessory nerve (CN XI) is exceedingly rare. We present the case of a 68-year-old male patient who presented with severe neck and shoulder wasting and a profound head drop. Despite the fact that standard conduction studies failed to demonstrate classic motor conduction blocks due to the highly proximal nature of the nerve involvement, a clinical diagnosis of MMN was suspected based on the asymmetric motor-only presentation, elevated Anti-GM1 antibodies, and steroid nonresponsiveness. The patient showed a robust and sustained functional recovery following treatment with intravenous immunoglobulin (IVIG), highlighting the importance of clinical judgment in atypical presentations of treatable neuropathies.

## Introduction

Multifocal motor neuropathy (MMN) is a chronic, immune-mediated neuropathy characterized by slowly progressive, asymmetric, predominantly distal limb weakness without objective sensory loss [1]. The classic disease phenotype predominantly affects the upper limbs, frequently presenting as a wrist drop or weakness in the small intrinsic hand muscles, making its initial manifestation mimic early-stage amyotrophic lateral sclerosis (ALS) or other motor neuron diseases.

The pathophysiology involves antibody-mediated dysfunction, frequently associated with IgM anti-GM1 antibodies, which leads to impaired nerve conduction without significant early axonal degeneration [2,3]. Standard diagnostic guidelines, such as those established by the European Academy of Neurology (EAN) and the Peripheral Nerve Society (PNS), rely heavily on electrophysiological proof of these persistent conduction blocks in noncompressive nerve segments [4]. According to these guidelines, this particular case can be classified as possible MMN. However, a significant subset of clinical variants presents without definitive conduction blocks due to deep-seated proximal segments or advanced axonal loss, creating a severe diagnostic dilemma [2]. Accurate differentiation is critical because MMN is highly treatable with intravenous immunoglobulin (IVIG), whereas it typically does not respond to, and may even be worsened by, corticosteroids [5,6].

While MMN predominantly targets the limbs, cranial nerve involvement is distinctly uncommon in clinical literature. When it does manifest, it typically involves the lower cranial nerves, presenting as transient lower facial weakness, hypoglossal palsy, or oculomotor disturbances late in the disease course [7]. In contrast, isolated or predominant presentation with a spinal accessory nerve (CN XI) palsy and accompanying pectoral nerve involvement is rarely reported. Anatomically, CN XI provides exclusive somatic motor innervation to the sternocleidomastoid and trapezius muscles, which are primarily responsible for neck rotation, lateral flexion, and shoulder shrugging.

When a patient presents with prominent, asymmetric weakness of these proximal cervical stabilizers, it frequently manifests clinically as a severe inability to maintain an upright head posture. Clinicians must systematically evaluate for structural cervical myelopathies, localized myopathies (such as isolated neck extensor myopathy), neuromuscular junction disorders (myasthenia gravis), or paraneoplastic syndromes [3]. The atypical topography in this case, involving concurrent CN XI palsy and pectoral nerve involvement, highlights a unique variant of MMN that expands the known clinical spectrum of dropped head syndrome and underscores the need for a high index of clinical suspicion when classic electrophysiological features are absent.

## Case presentation

The initial decline (2021-2022)

In April 2021, a 68-year-old retired customs officer presented with stiffness and pain in his posterior neck and shoulders, primarily on the right side. Initial MRI studies of the brain and cervical spine in June 2021 were largely unremarkable, showing only mild degenerative changes at C4-C5 without nerve root compression (Figure 1).

**Figure 1:**
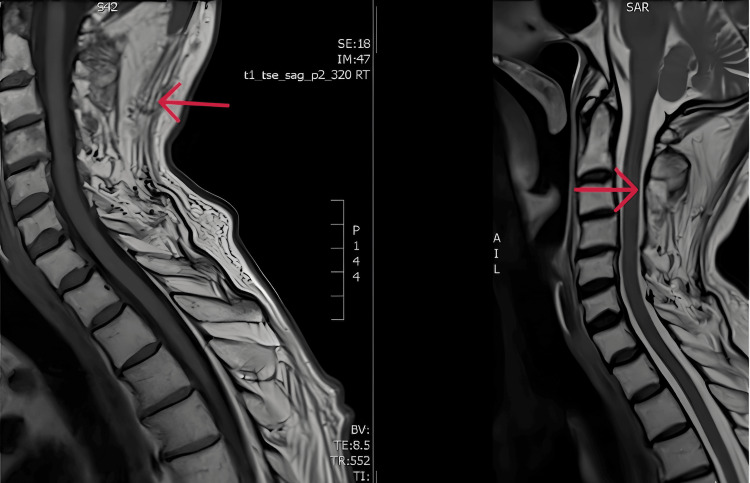
MRI cervical spine Arrows indicate regions of degenerative changes which are unrelated to the rest of the clinical picture

Over the following months, the patient developed a right shoulder drop and significant wasting of the shoulder girdle (Figures 2-3).

**Figure 2:**
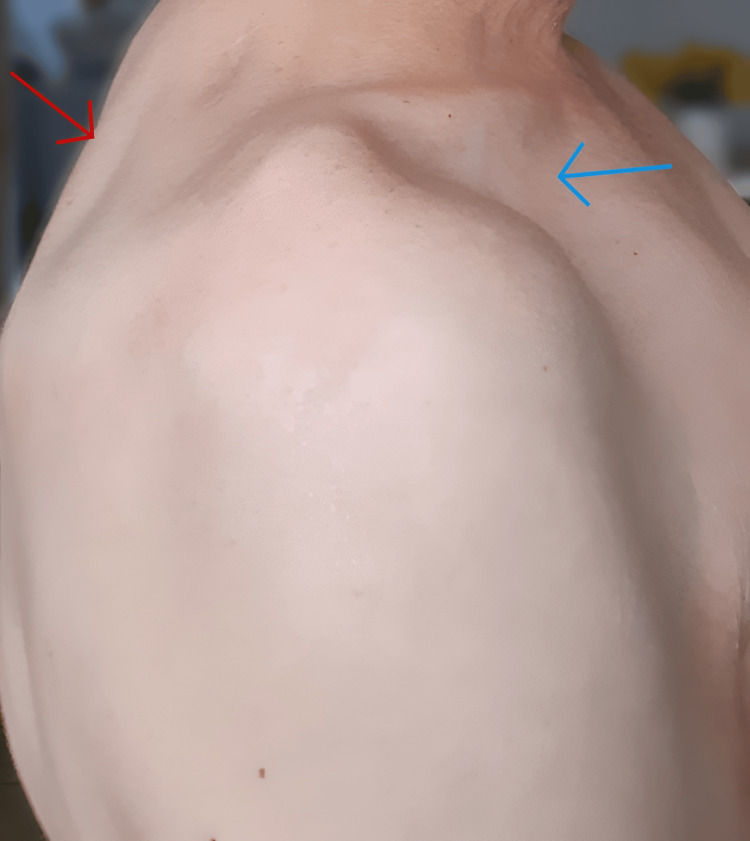
Examination findings (lateral view) Blue arrow shows clear wasting of the anterior chest muscles. Red arrow highlights significant wasting of the trapezius muscle

**Figure 3:**
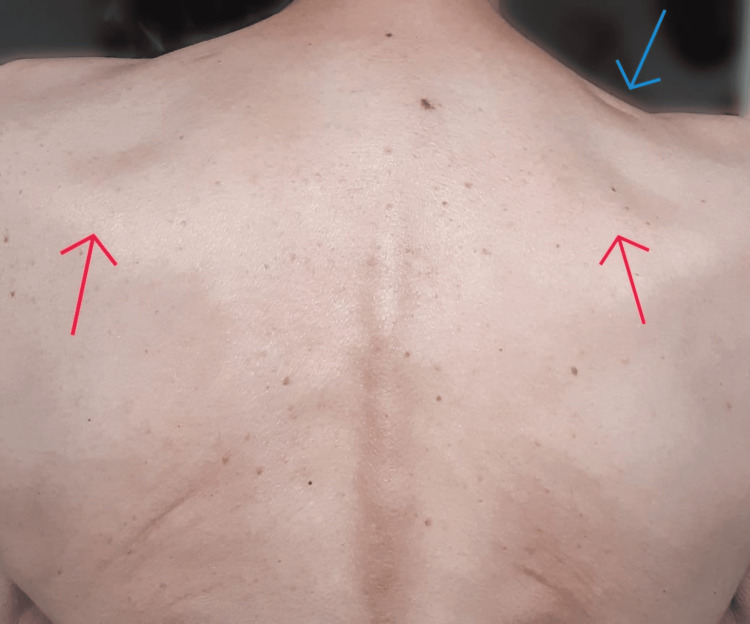
Examination findings (back) Blue arrow demonstrates a prominent right shoulder droop. The right shoulder sits noticeably lower than the left, indicating a loss of structural support or muscle tone on that side. Red arrows show bilateral trapezius muscle wasting, which is asymmetrical and more pronounced on the right side. This loss of muscle bulk contributes to the flattening of the upper back contour and exacerbates the downward droop of the right shoulder

By February 2022, investigations showed a raised creatine kinase (CK) of 948 U/L (Normal: 30-320 U/L), but inflammatory markers and autoantibodies were normal. When CK is elevated in MMN, it rarely reaches extremely high levels seen in primary muscle diseases. This elevation is generally caused by secondary muscle stress. Another possibility is malignancy-induced raised CK because tumor-related muscle breakdown can cause systemic leakage of CK into the bloodstream.

A diagnostic detour

In March 2022, a neck MRI revealed a suspicious finding in the left tonsil (Figure 4).

**Figure 4:**
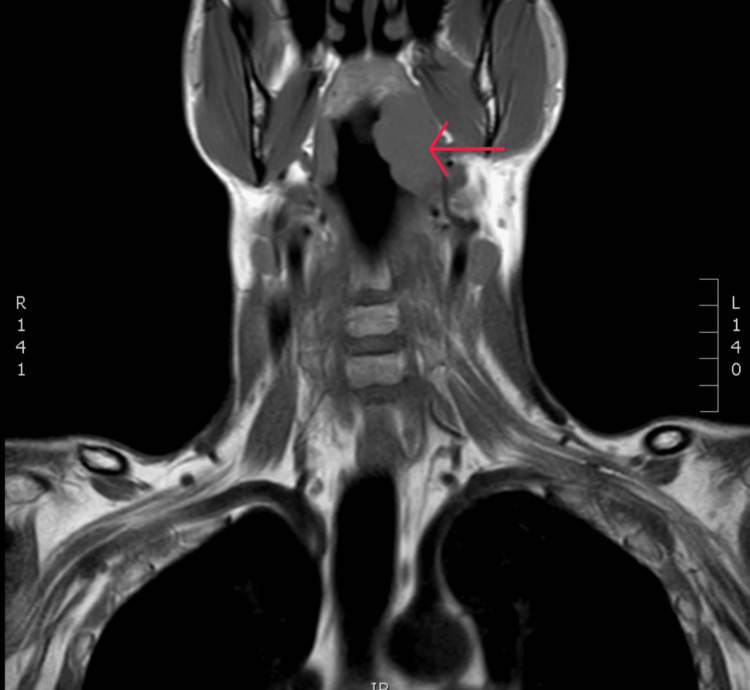
MRI neck MRI neck showed an enlarged tonsil (as indicated by arrow) which had no correlation to the patient's presenting neurological symptoms

A PET scan pointed toward squamous cell carcinoma (T4aN2BM0). Diagnosis was confirmed by biopsy of the tonsil in May 2022. The patient successfully completed chemoradiotherapy with cisplatin, achieving a complete response. Cisplatin neuropathy, radiation-induced plexopathy, paraneoplastic neuropathy, or local tumor-related nerve involvement were considered less likely than MMN, as the incidence of neurological symptoms did not align with the timeline of tumor identification and treatment. It wasn't local tumor-related nerve involvement, as a PET scan confirmed a left tonsillar mass with bilateral lymphadenopathy but no distant metastasis. Also, he had a left tonsillar mass, and the symptoms were worse on the right side. His neurological symptoms continued to decline despite the oncological recovery.

The neurological puzzle (2022-2023)

By late 2022, the patient was referred to neurology. Nerve conduction study (NCS) and electromyography (EMG) in April 2022 and July 2023 showed mixed chronic neurogenic and myogenic changes, particularly in the shoulder girdle and paraspinal muscles, but were not definitively diagnostic. The following are the results seen in 2023 (Tables 1-3).

**Table 1:** Nerve conduction studies (sensory nerve results) NCS: nerve conduction study Right upper limb NCS showing no sensory loss

Site	Latency (ms)	Latency (peak) (ms)	Amplitude (P-P) (µV)	Segment	Distance (mm)
Right median sensory					
Wrist-dig II	1.9	2.5	25.9	Wrist-Dig II	140
Right radial sensory					
Forearm-wrist	2.2	2.9	26.6	Forearm-Wrist	100
Right ulnar sensory					
Wrist-dig V	2.4	3.1	21.0	Wrist-Dig V	140

**Table 2:** Nerve conduction studies (motor nerve results) APB: abductor pollicis brevis; ADM: abductor digiti minimi Right distal segment nerve conduction studies failed to demonstrate classic motor conduction blocks due to the highly proximal nature of the nerve involvement

Site	Latency (ms)	Amplitude (mV)	Segment	Distance (mm)	CV (m/s)
Right median (APB) motor					
Wrist	3.7	6.8	-	-	-
Elbow	8.2	5.9	Elbow-wrist	250	56
Right ulnar (ADM) motor					
Wrist	2.7	8.4	-	-	-
Below elbow	7.2	7.9	Below elbow-wrist	245	54

**Table 3:** Electromyography EMG: electromyography Needle EMG shows pseudomyopathic changes (fibrillation potentials and positive sharp waves) possibly caused by chronic neurogenic remodeling, severe muscle fiber loss, and collateral reinnervation

Side	Muscle	Nerve	Nerve root	Insertional activity	Fibrillations	Positive sharp waves	Amplitude	Duration	Polyphasia	Recruitment	Interference pattern
Right	Deltoid	Axillary	C5-C6	Normal	1+ (mild)	Normal	Decreased	Normal	0 (None)	Rapid	Normal
Right	Infraspinatus	Suprascapular	C5-C6	Normal	Normal	Normal	Decreased	Normal	0 (None)	Rapid	Normal
Right	Biceps	Musculocutaneous	C5-C6	Normal	Normal	Normal	Decreased	Normal	0 (None)	Rapid	Normal
Right	First dorsal interosseous	Ulnar	C8-T1	Normal	Normal	Normal	Normal	Normal	0 (None)	Normal	Normal
Left	Deltoid	Axillary	C5-C6	Normal	Normal	2+ (moderate)	Decreased	Normal	0 (None)	Rapid	Normal
Left	Infraspinatus	Suprascapular	C5-C6	Normal	Normal	Normal	Decreased	Normal	0 (None)	Rapid	Normal
Left	Biceps	Musculocutaneous	C5-C6	Normal	Normal	Normal	Decreased	Normal	0 (None)	Rapid	Normal
Right	C7 paraspinal	Dorsal rami	C7	Normal	2+ (moderate)	2+ (moderate)	Not tested	Not tested	Not tested	Not tested	Not tested
Right	Vastus lateralis	Femoral	L2-L4	Normal	Normal	Normal	Decreased	Normal	0 (None)	Rapid	Normal
Right	Flexor digitorum profundus	Ulnar	C8-T1	Normal	Normal	Normal	Decreased	Normal	1+ (Mild)	Rapid	Normal

The EMG showed active denervation (fibrillation potentials and positive sharp waves) alongside small, short-duration motor unit potentials and early recruitment. While this looks like a muscle disease (myopathic pattern), it actually represents pseudomyopathic changes caused by chronic neurogenic remodeling, severe muscle fiber loss, and collateral reinnervation. In chronic motor neuropathies like MMN, this structural restructuring frequently mimics a primary myopathy. Therefore, an MMN diagnosis relies on the overall clinical picture and evidence of motor neuropathy with conduction blocks, rather than these isolated EMG features. However, this study was limited because key proximal muscles (pectoralis, trapezius, and sternocleidomastoid) were not examined.

Extensive blood work in 2022, including paraneoplastic screens (including Yo, Hu, Ri, Tr, anti-mitochondrial antibodies, CRMP 5, amphiphysin, Sox-1 antibodies, zinc 4 antibodies, titin and recoverin antibodies) and various antibodies including (anti-GM1 antibodies and anti-GQ1b antibodies), was negative. Therapeutic trials yielded no results. A trial of pyridostigmine for six months showed no response, and two separate trials of steroids (10 mg and 30 mg) failed to provide sustained improvement.

Peak symptomatic severity

By late 2023, the patient suffered from a profound "head drop," requiring hand support to keep his head upright. Examination revealed asymmetrical wasting of the bilateral trapezius (worse on the right), sternocleidomastoid, pectoralis major, and pectoralis minor. Power in neck extension was 1/5, and right shoulder elevation was 2/5. No fasciculations were noted, and cranial nerves 1-10 and 12 were intact. Repeat paraneoplastic antibody panel and ganglioside antibody testing were performed; on repeat analysis, anti-GM1 antibodies were elevated at 800 (reference range: 0-200), having previously been negative/unremarkable.

Treatment and outcome

In 2024, a therapeutic trial of IVIG was initiated at an induction dose of 2 g/kg administered over five days. Pre-assessment was done on September 30, 2024. The first IVIG dose was administered on December 9, 2024, and the first post-IVIG assessment was done on January 20, 2025. The patient demonstrated a significant clinical response across all quantitative neurofunctional parameters. The patient reported having functional improvement after two weeks of immunoglobulins.

Prior to the trial, his flaccid head drop was continuous, resulting in an unsupported head-holding time of zero seconds, which improved post-induction to two minutes of independent, upright hold. His functional mobility also showed marked acceleration. On manual muscle testing, objective motor recovery was highlighted by his neck extension power rising substantially from an initial 1/5 to a functional 3+/5.

Following this unambiguous clinical breakthrough, the patient transitioned to a long-term maintenance protocol consisting of 1 g/kg of IVIG infused every three months, which successfully facilitated sustained, cumulative functional restoration without any subsequent disease progression. By 2025, longitudinal reassessments confirmed that his independent head drop time had further increased to three minutes and 15 seconds, and his bilateral shoulder elevation power achieved complete normalization to a full 5/5 grading. Ultimately, his systemic neurological recovery culminated in an Overall Disability Sum Score (ODSS) of 0, indicating a total resolution of his functional disability and complete clinical stabilization. Although ODSS is not particularly sensitive to detecting head drop or shoulder girdle weakness, it was included to monitor for any involvement of other body regions over time. The patient reported functional status improvement, including improved neck extension and shoulder elevation after immunoglobulins, which was one of the tools used.

## Discussion

The diagnosis of MMN in this patient was supported by the clinical course and the presence of positive anti-GM1 antibodies, despite the absence of definitive electrophysiological findings [1,4]. The markedly asymmetric pattern of weakness, slowly progressive nature, and absence of sensory involvement raised strong suspicion for an immune-mediated motor neuropathy [2]. MMN remains a heavily challenging condition to identify, particularly when it deviates from its classical distal upper limb presentation. A critical diagnostic clue was elevated anti-GM1 antibodies and the lack of clinical improvement with corticosteroid therapy, which is characteristic of MMN [6,8]. The robust and sustained response to IVIG further favored the diagnosis [5,9]. While most immune-mediated inflammatory neuropathies (such as chronic inflammatory demyelinating polyradiculoneuropathy (CIDP)) or primary inflammatory myositis demonstrate a favorable therapeutic response to high-dose corticosteroids, MMN is distinctively nonresponsive to steroids. In fact, numerous reports indicate that corticosteroid administration can cause an acute, severe worsening of motor weakness in patients with MMN [5,6].

A central imperative in evaluating this case involves distinguishing this atypical presentation from several mimic syndromes, notably ALS and other motor neuron diseases. Because MMN presents with progressive, asymmetric, pure motor weakness, it is frequently misdiagnosed as early-stage flail arm syndrome or a localized lower motor neuron variant of ALS. However, the lack of upper motor neuron signs (such as hyperreflexia or spasticity), the complete absence of widespread fasciculations, the strict confinement of weakness to specific peripheral nerve territories (the spinal accessory and pectoral nerves), and the total stabilization and reversal of symptoms under immunomodulatory therapy definitively exclude a neurodegenerative motor neuron disease process.

An essential aspect of this case requires formally ruling out the patient's concurrent oncological diagnosis and treatment as causative or exacerbating factors for his neurological deficits. Cisplatin is heavily linked to severe peripheral neurotoxicity, which characteristically manifests as a symmetric, dose-dependent, sensory-predominant polyneuropathy. Furthermore, regional cervical external beam radiation can induce radiation-induced plexopathy or localized lower cranial nerve palsies due to chronic microvascular fibrosis. However, several key variables confidently exclude these therapies as alternative etiologies. Crucially, the patient’s neurological symptoms, including posterior neck pain, stiffness, and progressive shoulder dropping, began in April 2021, and a severe progressive decline was well-documented by November 2021. This clinical course preceded his oncological treatments, as his first chemoradiotherapy cycle was administered on May 31, 2022, and his last on July 11, 2022. Direct local tumor invasion or mechanical nerve compression by the malignancy can also be ruled out, as the diagnostic PET scan confirmed that the primary mass was restricted to the left tonsil with bilateral lymphadenopathy but no distant metastasis. Furthermore, while the malignant mass was located within the left tonsil, his neurological symptoms were markedly worse on the right side of his body. This anatomical misalignment, combined with the clear pre-existence of the neurological symptoms before any chemo- or radiotherapy exposures, definitively demonstrates that his cranial and pectoral nerve palsies arose from an independent, idiopathic neuropathic process rather than tumor mass effect, cisplatin neurotoxicity, or radiation-induced damage.

Similarly, local structural spinal disease and cervical radiculopathy were major initial diagnostic considerations given the prominent neck pain and subsequent flaccid head drop. Structural pathologies like severe cervical spondylosis, disk herniation, or foraminal stenosis can compress the cervical nerve roots or the upper spinal cord, leading to focal neurogenic wasting. While the patient’s initial cervical MRI showed mild degenerative changes at the C4-C5 level, it lacked any evidence of central canal stenosis, foraminal compromise, or spinal cord signal changes that could anatomically account for a bilateral CN XI palsy or chest wall wasting. Furthermore, a true cervical radiculopathy typically presents with objective dermatomal sensory loss, radiating dermatomal pain, and diminished deep tendon reflexes, none of which were observed in this patient.

This case is particularly notable as the clinical pattern was suggestive of isolated involvement of the CN XI and pectoral nerves, a presentation that is exceedingly rare in the literature [7]. A primary diagnostic hurdle in this case was the interpretation of the electrophysiological and serological testing. The absence of diagnostic motor conduction blocks on repeated NCS protocols initially argued against standard criteria for MMN. However, it is well-recognized that conventional surface nerve conduction studies possess technical limitations in detecting conduction blocks located within highly proximal nerve segments, such as the nerve roots, the brachial plexus, or the proximal trunk of cranial nerves. The active denervation and chronic neurogenic changes observed on his initial EMG, followed by the evolution of myogenic patterns in the severely wasted shoulder girdle, reflect a complex mixed pathology frequently encountered in chronic, advanced motor neuropathies where extensive axonal degeneration has occurred secondary to long-standing conduction block. This complex electrophysiological overlay can frequently mislead clinicians into making erroneous diagnoses of primary myopathies or anterior horn cell diseases. Furthermore, while serum IgM anti-GM1 antibodies are highly specific for MMN, they are absent in up to 30-50% of clinically confirmed cases, meaning a negative antibody screen never fully excludes the diagnosis.

Ultimately, the robust, rapid, and sustained therapeutic response to high-dose IVIG served as the definitive diagnostic validator. The patient demonstrated a marked, objective reversal of his flaccid head drop and a complete normalization of his upper limb functional scores within months of starting treatment, a response pattern that has been maintained over long-term follow-up. Because MMN is one of the very few chronic motor neuropathies that offers a potential for profound functional recovery [9,10], missing or delaying the diagnosis can lead to irreversible axonal dropout and permanent muscle atrophy [3,4]. This case emphasizes integrating clinical judgment with therapeutic response. Early initiation of appropriate therapy is crucial to prevent progression and long-term disability [1].

## Conclusions

This case highlights an uncommon proximal presentation of MMN with asymmetric neck and shoulder weakness (by isolated involvement of the CN XI and pectoral nerves), elevated anti-GM1 antibodies, and nondiagnostic electrophysiological studies. Such rare manifestations can easily be misdiagnosed as structural spine disease or more progressive neurodegenerative conditions like motor neuron disease. Although conduction block is considered a hallmark of MMN, it may not be demonstrable in all patients, particularly when proximal nerve segments are preferentially involved. Routine nerve conduction studies may inadequately assess proximal structures, reducing the sensitivity of EMG in atypical presentations such as this one. To bridge this diagnostic gap in suspected proximal MMN variants, clinicians should proactively utilize advanced diagnostic modalities. This includes performing targeted needle EMG of clinically affected proximal muscles alongside advanced neuroimaging such as brachial plexus MRI or high-resolution MR neurography to detect proximal nerve hypertrophy or increased T2 signal. Additionally, proximal stimulation techniques, such as electrical or magnetic root stimulation, should be utilized to evaluate nerve segments that remain inaccessible to conventional nerve conduction setups. Clinicians must remain vigilant: when presented with asymmetric, pure motor weakness that fails to respond to corticosteroids, a therapeutic trial of IVIG should be considered after exclusion of structural, malignant, paraneoplastic, radiation-induced, and motor neuron disease mimics, preferably under neuromuscular specialist supervision. Because MMN remains a treatable condition, bridging the gap between atypical clinical signs and early initiation of therapy can fundamentally change a patient's trajectory, preventing muscle wasting and long-term disability while facilitating significant functional recovery.
